# Relationships between cocoa mass percentage, surface color, free phenolic compounds content and antioxidant capacity of commercially available dark chocolate bars

**DOI:** 10.1007/s13197-020-04898-1

**Published:** 2020-11-27

**Authors:** Natalia Mikołajczak, Małgorzata Tańska

**Affiliations:** Department of Food Plant Chemistry and Processing, Faculty of Food Sciences, University of Warmia and Mazury, Plac Cieszyński 1, 10-726 Olsztyn, Poland

**Keywords:** Chocolate bar, Color, Phenolic compounds, Antioxidant capacity, Cocoa mass content

## Abstract

The aim of the study was to assess the relationships between cocoa mass percentage declared by producer and color, free phenolic compounds content and antioxidant capacity of chocolate bars. The research materials were commercially available 2 dessert (with 30 and 50% of cocoa mass) and 10 bitter chocolate bars (with 40–90% of cocoa mass). The scope of analysis included determining chocolate bars surface color using digital image analysis, content of free phenolic compounds (total, flavonoids and proanthocyanidins) using spectrophotometric methods and antioxidant capacity using the Cuprac method.

Based on the results, it was generally found that bitter chocolate bars were characterized by a darker color and a higher content of free phenolic compounds (252.38–703.13 mg/100 g), including flavonoids (29.01–89.55 mg/100 g) and proanthocyanidins (52.23–224.47 mg/100 g), compared to dessert chocolate bars (241.70, 38.58 and 58.99 mg/100 g on average, respectively). The study showed that the cocoa mass content in the chocolate bars was strongly positively correlated with the phenolic compounds content (in particular flavonoids) and the antioxidant capacity. On the other hand, these properties of the chocolate bars were less dependent on the surface color.

## Introduction

In recent years, an increase in the incidence of diseases such as obesity and overweight has been observed among the population. It was proven that these diseases are caused by low physical activity and excessive consumption of sweets (high-energy products with low nutritional value). In a wide range of confectionery products there are also products that should not be excluded from the daily diet (subjected to moderate consumption) due to the presence of compounds that have a positive effect on the consumer's health, e.g. polyphenols, minerals, vitamins, and oligosaccharides (Nichols and Katiyar 2010). The example of this may be some chocolate products (Rogovska and Čukanová 2015).

According to the Directive (2000/36/WE) of the European Parliament and of the Council of 23 June 2000, chocolate is defined as 'the product obtained from cocoa products, and sugars, which contains not less than 35% total dry cocoa solids, including not less than 18% cocoa butter and no less than 14% dry non-fat cocoa mass'. This definition specifies that the cocoa mass may include cocoa liquor and/or cocoa powder and cocoa butter. Cocoa liquor and cocoa powder are characterized by a high content of phenolic compounds, which give chocolate a specific bitterness and astringency, as well as aroma and color (Elwers et al. 2009). In addition, the phenolic compounds are responsible for preventing the development of chronic non-communicable diseases, i.a. cancers, diseases of the cardiovascular or nervous systems, increase resistance to coronary and heart disorders, prevent platelet aggregation (D’Archivio et al. 2007), stimulate hormone production, exert a positive effect on lipid metabolism, and are also involved in several other biological functions, such as skin protection (Nichols and Katiyar 2010). The dominant group of phenolic compounds in chocolate are flavonoids, in particular proanthocyanidins, catechins and quercetin, which may constitute up to 90% of the total phenolic compounds in chocolates with a high content of cocoa mass (Wollgast and Anklam 2000).

The main factor differentiating chocolate bars available on the market, is the content of cocoa mass, which must be declared by the producer on the packaging label (Yates 2009). In Poland, generally the highest cocoa mass content is found in bitter chocolate bars (usually above 75%), less cocoa mass occurs in dessert chocolate bars (30–70%), while in milk chocolate bars it is only 25%. The products also differ in the content of sugar which the largest amounts are found in milk chocolate bars (over 50%), lower amounts in dessert chocolate bars (40–50%) and bitter chocolate bars (up to 40%) (Jabłońska-Ryś 2012). In some countries, e.g. the USA, dark chocolate is a category of chocolate that includes semisweet and bittersweet chocolates. Typically, semisweet chocolate contains 35–45% cacao and is sweeter, while bittersweet chocolate usually contains at least 50% cacao (NCA 2019). However, there are no legal regulations to distinguish chocolate bars between dark, bittersweet and semisweet.

The most popular among consumers are milk and filled chocolate bars. However, more and more often they also choose the dark chocolate bars (dessert and bitter) classified as functional food whose purpose is to improve consumer health and well-being (Rogovska and Čukanová 2015). There are a number of studies on the phenolic compounds in chocolate products (Belŝĉak et al. 2009; Albak and Tekin 2016; Gu et al. 2006; Pavlović et al. 2017), while information on the relation between the phenolic compounds content and the cocoa mass percentage in the products is rather scarce. Likewise, antioxidant capacity of chocolate was also previously studied (Miller et al. 2006; Baceral Leite et al. 2013; Brcanović et al. 2013; Laličić-Petronijević et al. 2016). However, most of the studies were focused on effect of process and storage conditions on chocolate composition (Albak and Tekin 2016; Laličić-Petronijević et al. 2016) and on comparison of various chocolate types (Miller et al. 2006; Belŝĉak et al. 2009; Meng et al. 2009). Despite the valuable data on the variability among chocolate types in phenolic compounds content and antioxidant capacity, there is still a lack of knowledge on the relation between these properties of commercially available dark chocolate bars and information that is readily accessible to the consumer (color and cocoa mass content).

The aim of the research was to determine the impact of the minimum content of dry cocoa solids, declared by the producer, in dessert and bitter chocolate bars for color, free phenolic compounds content and antioxidant capacity. In addition, the occurrence of correlations between the analyzed properties was also evaluated.

## Material and methods

### Chemicals and plant materials

n-hexane, acetone, Folin-Ciocalteu reagent, anhydrous sodium carbonate, sodium nitrate, aluminum (III) chloride, sodium hydroxide, 1-butanol, copper chloride, neocuproin, ammonium acetate buffer and D-catechin were purchased from Sigma (Sigma-Aldrich, USA).

The research materials were 12 popular chocolate bars produced and bought in Poland, including 2 dessert chocolate bars and 10 bitter chocolate bars. All types of chocolate bars are available on the European food market (the same name and composition), and differed in the percentage content of dry cocoa solids in the product declared by the producer on the packaging label by the use of the term 'cocoa mass minimum … %' or 'cocoa content: minimum … '. Three packages of each type of chocolate bars from three different batches were purchased for the research. The research material characteristics including the type of chocolate bar, cocoa mass content and composition is presented in Table 1.

**Table 1:** Chocolate bars (CB) used in the research and their color parameters measured by the image analysis

Chocolate bar symbol	Chocolate bar type	Cocoa mass/cocoa [%]*	Composition (declared on the packaging)	H (Hue) [^o^]		S (Saturation) [%]		I (Intensity) [%]	
				${{\bar{x}}}$	δ	${{\bar{x}}}$	$\delta$	${{\bar{x}}}$	$\delta$
CB1	dessert	30 (cocoa)	Sugar, cocoa liquor, defatted milk powder, milk fat, whey powder, emulsifier (from soybean E322), flavor (vanillin)	18.33^d^	0.58	7.55^c^	0.21	2.98^e^	0.12
CB2	bitter	40 (cocoa mass)	Cocoa liquor, sugar, cocoa butter, emulsifier: lecithin (from soy), natural vanilla extract	21.67^c^	1.53	8.46^b,c^	0.56	3.31^b,c,d,e^	0.13
CB3	bitter	45 (cocoa mass)	Sugar, cocoa liquor, plant fat (palm oil, shea), cocoa butter, emulsifiers: lecithin (from soy) and E476, flavor	21.67^c^	0.58	8.58^b^	0.37	3.31^b,c,d,e^	0.15
CB4	dessert	50 (cocoa mass)	Sugar, cocoa liquor, reduced-fat cocoa, cocoa butter, milk fat, emulsifiers (soya lecithin, E476), salt, aroma	20.67^c,d^	0.58	8.06^b,c^	0.17	3.15^c,d,e^	0.19
CB5	bitter	51 (cocoa)	Maltitol 48%, cocoa liquor, cocoa butter, inulin, soy lecithin—emulsifier, vanillin—aroma	19.33^c,d^	0.58	7.94^b,c^	0.28	3.12^d,e^	0.09
CB6	bitter	55 (cocoa mass)	Cocoa liquor, sugar, cocoa butter, reduced-fat cocoa, emulsifier (soya lecithin)	21.33^c^	0.58	8.20^b,c^	0.22	3.23^c,d,e^	0.18
CB7	bitter	64 (cocoa mass)	Cocoa liquor, sugar, reduced-fat cocoa, cocoa butter, emulsifiers (soya lecithin and E476), flavor	21.00^c^	0.00	8.22^b,c^	0.22	3.30^b,c,d,e^	0.23
CB8	extra bitter	65 (cocoa mass)	Cocoa liquor, sugar, cocoa butter, soy lecithin (E322), aroma, whole milk powder	24.67^a,b^	0.58	9.57^a^	0.35	3.60^b,c,d^	0.18
CB9	bitter	70 (cocoa mass)	Cocoa liquor, sugar, reduced-fat cocoa, cocoa butter, emulsifier: lecithin (from soy), flavor	21.33^c^	1.53	8.38^b,c^	0.17	3.36^b,c,d,e^	0.25
CB10	bitter	72 (cocoa mass)	Cocoa liquor, sugar, cocoa butter, emulsifier: lecithin (soy), vanilla extract	24.67^a,b^	0.58	9.77^a^	0.11	3.74^a,b^	0.09
CB11	bitter	76 (cocoa mass)	Cocoa liquor, sugar, cocoa butter,, reduced-fat cocoa, emulsifier (soya lecithin)	24.33^b^	0.58	9.62^a^	0.33	3.64^a,b,c^	0.20
CB12	bitter	90 (cocoa mass)	Cocoa liquor, reduced-fat cocoa, sugar, cocoa butter, emulsifiers: lecithin (from soya) and E476, flavor	27.00^a^	1.00	10.44^a^	0.50	4.10^a^	0.15

^*^ Minimum content of cocoa mass/cocoa (declared on the packaging); ${\mathbf{\bar{x}}}$–average value; —standard deviation; a,b,c, … – average values in columns with the same letter are not significantly different at *p* ≤ 0.05

## Determination of surface color using digital image analysis (DIA)

The color of chocolate bars was measured according to the methodology of Tańska et al. (2011) using a Nikon DXM-1200 (Nikon Instruments, Melville, USA) charge coupled device (CCD). The color of chocolate bars was expressed in HSI color space.

## Preparation of chocolate bar extracts

Preparation of extracts was carried out according to the method described by Meng et al. (2009). Defatting of chocolate bars (5 g) was carried out by twofold extraction with n-hexane (10 mL). A non-fat mass was dried under natural conditions, i.e. air at 20 °C for 24 h. The extracts were prepared from the non-fat mass (2 g) using twofold extraction (10 mL) with 80% acetone, and then evaporated to dryness at temperature below 50 °C in a vacuum evaporator (Büchi, type R-210; Büchi Labortechnik, Flawil, Switzerland). The residue was dissolved in pure methanol (25 mL) and used to determine the content of free phenolic compounds and the antioxidant capacity in chocolate bars.

## Determination of free phenolic compounds content

The content of total free phenolic compounds and free flavonoids were determined according to the method described by Meng et al. (2009). A color reaction with Folin-Ciocalteu reagent (total phenolic compounds content) and with 5% aqueous sodium nitrate solution and 10% aqueous aluminum (III) solution (flavonoids content) were performed. Absorbance was measured at 720 nm (total free phenolic compounds) and 510 nm (free flavonoids) using a Unicam UV/Vis UV2 spectrophotometer (ATI Unicam, Cambridge, UK). Free proanthocyanidins content was determined according to the method described by Belŝĉak et al. (2009). A reaction with 1-butanol-HCl reagent (0.7 g of iron reagent (FeCl_3_ × 7 H_2_O) was added to 25 mL of concentrated HCl and then the solution was made up with a 1-butanol to a volume of 1000 mL) in a boiling water bath for 2 h was performed. After the solution cooling down to room temperature, absorbance at 550 nm was measured using the Unicam UV/Vis UV2 spectrophotometer.

The total free phenolic compounds, free flavonoids and free proanthocyanidins contents were calculated based on the D-catechin calibration curve and expressed as mg D-catechin in 100 g of product.

## Determination of antioxidant capacity

The antioxidant capacity of chocolate bars was determined using the Cuprac method described in the work of Apak et al. (2008). The extract (0.2 mL) was evaporated under vacuum in a Büchi rotary evaporator R-210 type and 1 mL of copper chloride, neocuproin, ammonium acetate buffer and 0.9 mL of distilled water were added. After 30 min, the absorbance at 450 nm was measured using the Unicam UV/Vis UV2 spectrophotometer. The antioxidant capacity was expressed as mM Trolox (TE) per 100 g of chocolate bar.

## Statistical analysis

All analyzes were performed in triplicate and the obtained results were analyzed statistically using the Statistica 12.5 PL (StatSoft, Cracow, Poland). The differences between the chocolate bars was assessed using a variance analysis (ANOVA) at the significance level of *p*  ≤ 0.05 and Tukey's test. The intra‐sample quality variation of the chocolate bars was assayed using principle component analysis (PCA) at *p*  ≤ 0.05 significance level.

## Results and discussion

### Color of chocolate bars

Bitter chocolate bars in comparison to dessert chocolate bars were characterized by higher values of individual components of the HSI color space (Table 1). The highest values of individual parameters H (Hue), S (Saturation) and I (Intensity) were found for bitter chocolate bar CB12 (cocoa mass content was at least 90%), and amounted to 27.00º, 10.44% and 4.10%, respectively. Other bitter chocolate bars (with a minimum of 40–70% cocoa mass/cocoa in the product) were characterized by the values of these components in the ranges: 19.33–24.67º, 7.94–9.77%, and 3.12–3.74%, respectively. The lowest values of the color components characterized dessert chocolate bar CB1 with the lowest cocoa content (about 30% in the product). The color of this chocolate bar, compared to the others, was more red (H = 18.33º) with a low saturation (S = 7.55%) and intensity (I = 2.98%). The greater share of redness in the color of chocolate bar with low cocoa mass content (28.77%) compared to chocolate bars with its higher content (49.77% and 64.27%) were also indicated by López-Hernández and Quintero-Cerón (2016). However, there is a lack of literature data on the color parameters of chocolates with various cocoa mass content.

## Content of free phenolic compounds in chocolate bars

The content of free phenolic compounds in studied chocolate bars is presented in Table 2. Bitter chocolate bars with a minimum of 40−90% content of cocoa mass/cocoa, were characterized by the high total content of free phenolic compounds, 252.38–703.13 mg/100 g of the product. Dessert chocolate bars with at least a 30% content of cocoa mass (CB1) contained slightly less free phenolic compounds, 139.03 mg/100 g. Similar results of phenolic compounds in dark chocolate bars were presented by Pimentel et al. (2010). In their study, the chocolate bar with a 40% content of cocoa mass had the content of phenolic compounds of 499.00 mg/100 g, while in chocolate bar with a 71% content of the cocoa mass it was almost 1.3-fold higher. Baceral Leite et al. (2013) found that the content of phenolic compounds in dark chocolate bars (with a 70% content of cocoa mass) made from cocoa beans grown in Brazil, is 154.6–91.1 mg in 100 g of product. Cooper et al. (2008) analyzing 46 plain dark chocolate bars (with the content of cocoa mass in the range of 34−100%) purchased in Europe, estimated that the total content of phenolic compounds is in the range from 340 to 2,340 mg/100 g.

**Table 2:** Content of free phenolic compounds and their antioxidant capacity in analyzed chocolate bars

Chocolate bar symbol	Total free phenolic compounds [mg/100 g]		Free flavonoids [mg/100 g]			Free proanthocyanidins [mg/100 g]			Antioxidant capacity [mM TE/100 g]	
	${{\bar{x}}}$	δ	${\bar{x}}$	δ	[%]*	${{\bar{x}}}$	δ	[%]*	${{\bar{x}}}$	δ
CB1	139.03^j^	17.92	24.41^g^	0.31	17.56	28.48^i^	1.14	20.48	17.58^e,f^	0.73
CB2	252.38^i^	6.73	37.63^f^	3.75	14.91	52.23^h^	4.00	20.69	15.30^g^	0.07
CB3	321.63^h^	14.84	29.01^g^	0.16	9.02	84.48^g^	0.61	26.27	16.18^f,g^	0.12
CB4	344.37^g,h^	13.65	52.74^d,e^	2.19	15.31	89.49^g^	1.72	25.99	17.31^e,f^	0.83
CB5	355.94^g^	9.39	37.21^f^	2.66	10.45	148.55^d^	5.55	41.74	18.07^d,e^	0.18
CB6	414.04^f^	15.74	46.16^e^	2.97	11.15	117.62^f^	1.62	28.41	19.59^b,c,d^	0.83
CB7	480.64^e^	8.34	58.16^c,d^	0.16	12.10	205.44^b^	7.27	42.74	19.31^c,d^	0.87
CB8	666.45^b^	6.86	65.36^b,c^	2.81	9.81	212.08^a,b^	8.42	31.82	21.30^b^	0.59
CB9	514.61^d^	5.00	50.26^e^	2.03	9.77	177.16^c^	1.14	34.43	21.01^b,c^	0.26
CB10	703.13^a^	7.33	62.92^c^	1.40	8.95	223.71^a^	8.74	31.82	21.26^b^	0.10
CB11	588.48^c^	8.92	71.43^b^	5.01	12.14	224.47^a^	9.85	38.14	20.31^b,c^	0.92
CB12	618.84^c^	9.19	89.55^a^	3.09	14.47	212.48^a,b^	8.19	34.34	28.79^a^	0.28

[%]* – % of the total content of free phenolic compounds, ${{\bar{x}}}$ – average value; δ — standard deviation;

a,b,c, …. – average values in columns with the same letter are not significantly different at *p* ≤ 0.05

Symbols of chocolate bars are explained in Table 1

The content of free flavonoids in the analyzed chocolate bars was significantly different (*p* ≤ 0.05) and was in the range of 24.41–89.55 mg/100 g of the product. The lowest amount was found in dessert chocolate bar CB1 (with at least a 30% content of cocoa), and the highest in bitter chocolate bar CB12 (with a minimum of 90% cocoa mass). The bitter chocolate bars (with a minimum content of cocoa mass/cocoa in the range of 40–90%) were characterized by a free flavonoids content of 29.01–89.55 mg/100 g, while the dessert chocolate bar with at least a 50% cocoa mass (CB4) contained 52.74 mg flavonoids/100 g. Furthermore, it was found that the dessert chocolate bars had a higher percentage share of free flavonoids in the total content of free phenolic compounds compared to the bitter chocolate bars (15.31–17.56% vs. 8.95–14.91%). Meng et al. (2009) showed that the bitter chocolate bars contained 28.30 mg flavonoids in 100 g. However, in the cited work the content of cocoa mass in the products was not specified. In turn, Brcanović et al. (2013) reported that all flavonoids can constitute up to 37–70% of total phenolic compounds in dark chocolate bars.

The lowest content of free proanthocyanidins, 28.48 and 52.23 mg/100 g, was found in chocolate bars CB1 (dessert) and CB2 (bitter). These products were also characterized by the lowest, declared by the producer, minimum content of cocoa mass/cocoa in the product, 30 and 40%, respectively. Chocolate bars CB3 and CB4 (the minimum content of cocoa mass was 45% and 50%, respectively) were characterized by a higher content of free proanthocyanidins, 84.48 and 89.49 mg/100 g, respectively. Bitter chocolate bars with over 50% content of cocoa mass/cocoa were characterized by free proanthocyanidins in the range of 117.62 to 224.47 mg/100 g. The highest content of these compounds was found in bitter chocolate bar CB11 (76% of cocoa mass content). It was found that the free proanthocyanidins in the analyzed chocolate bars accounted for 20.48–42.74% of all free phenolic compounds. The dark chocolate bars analyzed by Hii et al. (2009) contained proanthocyanidins in the amount of 278–410 mg/100 g. However, surprisingly high content of proanthocyanidins was determined in the work of Gu et al. (2006). They found that in the dark chocolate bars the content of proanthocyanidins reached even 2,000 mg in 100 g of the product.

In this study, it was observed that the content of total free phenolic compounds, flavonoids and proanthocyanidins strongly dependent on cocoa mass/cocoa in the product (correlation coefficient (r) in the range of 0.901–0.924). Also the content of free proanthocyanidins was strongly positively correlated with the total free phenolic compounds (r = 0.943). Laličić-Petronijević et al. (2016) and Meng et al. (2009) also showed a strong correlation between total phenolic compounds content and the flavonoids content (r = 0.998 and 0.930, respectively), which clearly indicates flavonoids as the main group of chocolate polyphenols. Lie et al. (2014) observed a negative correlation between the L*, a* and b* components and the total polyphenols content in the cocoa powder. According to the research of these authors, the darker color of the cocoa powder does not indicate higher total phenolic compounds content.

## Antioxidant capacity of chocolate bars

All analyzed chocolate bars were characterized by high and significantly differentiated (*p* ≤ 0.05) antioxidant capacity (Table 2). Dessert chocolate bars were characterized by antioxidant capacity in the range of 17.31–17.58 mM TE/100 g, while bitter chocolate bars in the range of 15.30–28.79 mM TE/100 g. The highest antioxidant capacity was found in bitter chocolate bars with a high content of cocoa mass (minimum 65–90%), and the lowest in bitter chocolate bars CB2 and CB3, in which the minimum content of cocoa mass was 40 and 45%, respectively. Similarly, in research conducted by Meng et al. (2009), dark chocolate bars showed the highest antioxidant capacity, followed by milk and white chocolate bars.

Taking into account relationships between analyzed parameters of chocolate bars, it was found that the antioxidant capacity correlated mostly positively with the content of cocoa mass/cocoa in the product (r = 0.856), the content of free flavonoids (r = 0.833) and individual components of the HSI color space (r in the range of 0.785–0.797). Laličić-Petronijević et al. (2016) reported that the antioxidant capacity of chocolate bars determined using various assays is strongly correlated with the content of proanthocyanidins (r ≥ 0.966) and flavonoids (r ≥ 0.948). In turn, Brcanović et al. (2013) indicated proanthocyanidins as the main phenolic compounds determining the antioxidant capacity of chocolate bars.

## PCA model for chocolate bars

**Fig. 1:**
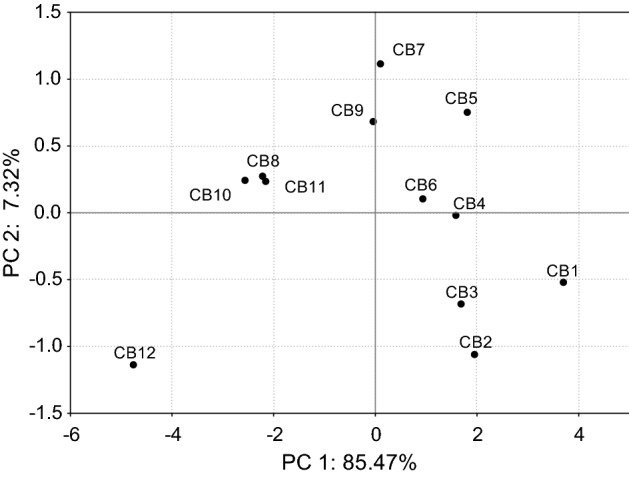
Projection of the studied chocolate bars in the space defined by the first two principal components (PC1 and PC2) based on the determined parameters. Symbols of chocolate bars are explained in Table 1

The PCA analysis revealed variations among studied chocolate bars (Fig. 1). According to the first two principal components (PC1 and PC2) that accounted for 92.59% of total variance, the chocolate bar with the highest cocoa mass content (CB12) clearly differed from the others. The product characterized by the most red, saturated and intense color, and at the same was the most abundant in flavonoids. In turn, the chocolate bars with 30–45% of cocoa mass (CB1–CB3) contained the lowest amount of all polyphenols from all analyzed chocolate bars (less than 320.00 mg/100 g), what also resulted in visible separation of these samples on PCA scores plot.

## Conclusions

In the present study the minimum content of dry cocoa solids in chocolate bars, which was declared on the packaging label, have been taken into account. Based on the value of correlation coefficients, it can be concluded that the content of cocoa mass and the color of chocolate bars may be preliminary information for the consumer on the content of phenolic compounds, mainly flavonoids, in the product. The obtained results confirm that chocolate bars, especially with high cocoa mass content, are rich in free phenolic compounds, including flavonoids and proanthocyanidins. However, there are deviations from these dependencies, which may result from partially overlapping ranges of cocoa mass content typical for bitter and dessert chocolate bars. Furthermore, the producers provide a minimum, not exact, content of cocoa mass in chocolate bars. Also the products may contain not typical components, e.g. milk powder in bitter chocolate bars and plant fat (palm, shea) in a larger amount than cocoa butter in dark chocolate. These ingredients can also affect the physicochemical characteristics of chocolate bars.
